# Keys to success of a community of clinical practice in primary care: a qualitative evaluation of the ECOPIH project

**DOI:** 10.1186/s12875-018-0739-0

**Published:** 2018-05-09

**Authors:** David Lacasta Tintorer, Josep Maria Manresa Domínguez, Enriqueta Pujol-Rivera, Souhel Flayeh Beneyto, Xavier Mundet Tuduri, Francesc Saigí-Rubió

**Affiliations:** 1 Centre d′Atenció Primària Gran Sol, Gerència d’Àmbit d’Atenció Primària Metropolitana Nord, Institut Català de la Salut, Avinguda del Doctor Bassols, 112 - 130, 08914 Badalona, Spain; 2 Unitat de Suport a la Recerca Metropolitana Nord, IDIAP Jordi Gol. CAP El Maresme, Camí del Mig, 36 planta 4a, 08303 Mataró, Spain; 3 Universitat Autònoma de Barcelona, Plaça Cívica, s/n, 08193 Bellaterra, Cerdanyola del Vallès, Spain; 4 Institut Universitari d’Investigació en Atenció Primària (IDIAP Jordi Gol), Gran Via Corts Catalanes, 587, àtic, 08007 Barcelona, Spain; 5 Unitat de Suport a la Recerca Barcelona Ciutat, IDIAP Jordi Gol, Carrer Sardenya 375, 08025 Barcelona, Spain; 6 Faculty of Health Sciences, Universitat Oberta de Catalunya, Barcelona. Av. Tibidabo, 39-43, 08035 Barcelona, Spain

**Keywords:** Primary health care, Problem solving, Telemedicine, Referral and consultation, Education medical continuing

## Abstract

**Background:**

The current reality of primary care (PC) makes it essential to have telemedicine systems available to facilitate communication between care levels. Communities of practice have great potential in terms of care and education, and that is why the Online Communication Tool between Primary and Hospital Care was created. This tool enables PC and non-GP specialist care (SC) professionals to raise clinical cases for consultation and to share information. The objective of this article is to explore healthcare professionals’ views on communities of clinical practice (CoCPs) and the changes that need to be made in an uncontrolled real-life setting after more than two years of use.

**Methods:**

A descriptive-interpretative qualitative study was conducted on a total of 29 healthcare professionals who were users and non-users of a CoCP using 2 focus groups, 3 triangular groups and 5 individual interviews. There were 18 women, 21 physicians and 8 nurses. Of the interviewees, 21 were PC professionals, 24 were users of a CoCP and 7 held managerial positions.

**Results:**

For a system of communication between PC and SC to become a tool that is habitually used and very useful, the interviewees considered that it would have to be able to find quick, effective solutions to the queries raised, based on up-to-date information that is directly applicable to daily clinical practice. Contact should be virtual – and probably collaborative – via a platform integrated into their habitual workstations and led by PC professionals. Organisational changes should be implemented to enable users to have more time in their working day to spend on the tool, and professionals should have a proactive attitude in order to make the most if its potential. It is also important to make certain technological changes, basically aimed at improving the tool’s accessibility, by integrating it into habitual clinical workstations.

**Conclusions:**

The collaborative tool that provides reliable, up-to-date information that is highly transferrable to clinical practice is valued for its effectiveness, efficiency and educational capacity. In order to make the most of its potential in terms of care and education*,* organisational changes and techniques are required to foster greater use.

**Electronic supplementary material:**

The online version of this article (10.1186/s12875-018-0739-0) contains supplementary material, which is available to authorized users.

## Background

A characteristic feature of primary (PC) surgeries is that they have to attend to a high number of patients suffering from many different health problems, whose clinical complexity is considerable [1, 2]. This means that physicians have to deal with several aspects at once, which may raise a multitude of issues in day-to-day clinical practice [3–6]. That is why such professionals require an effective system to search for and find information that enables them not only to update their knowledge, but also to solve problems efficiently and effectively [7–9].

Clinical sessions and individual conversations (in person and over the phone) with non-GP specialist care (SC) professionals are options that allow them to resolve such issues. However, given that the health system is at saturation point, communication between PC and SC is not easy, quick or effective, and it leads to many referrals to SC (hospitalisation or specialist outpatient clinics) that generally entail excessive delays for appointments [2, 10].

Despite the increase in access to electronic sources of information, PC physicians usually raise their queries with other colleagues in the first instance, resorting to the Internet as the second option [8, 11–13]. Several experiences that make the most of the advantages that telemedicine offers with respect to improving communication between PC and SC have proved to be beneficial in terms of efficiency, cost-effectiveness and improved medical care [14], with a high degree of satisfaction [15–17].

One of the latest approaches is the creation of communities of practice (CoPs) [18]. Applied to the field of healthcare, communities of clinical practice (CoCPs) are online platforms that draw on the advantages of Web 2.0 for the creation, dissemination and management of clinical knowledge by and among healthcare professionals working at different levels of care [19]. While evidence of their usefulness is still somewhat limited, these virtual communities have been shown to have not only a considerable capacity to transfer knowledge acquired in daily clinical practice [20–22], but also great educational potential [23–28].

While most studies have focused on analysing the results of CoCP use [29–31], on the promotion of evidence-based clinical practice [32, 33] and on final decision-making [34], it is crucial to consider the determinants of the use of CoCPs in order to fully understand the use thereof. In other words, it is necessary to perform an ex-ante analysis in the study of the determinants of COCP use instead of an ex-post analysis of the determinants of the results of CoCP use. Thus, this article presents an ex-ante analysis and aims to provide evidence of the determinants of CoCP use, beyond the study of the results thereof.

A CoCP called the ECOPIH was created in 2009. The abbreviation stands for *Eina de Comunicació Online entre Primària i Hospitalària* in the Catalan language, or Online Communication Tool between Primary and Hospital Care in English. It is a CoCP that uses a Web 2.0 platform for communication between PC and SC, bringing together healthcare professionals from PC centres and non-GP specialists from several hospitals in Badalona and Sant Adrià de Besòs (two cities in the Barcelona metropolitan area, Spain) [35]. The study of a CoCP after several years should enable an assessment to be made of whether it has met this need, by analysing the strengths and weakness that determine its use and identifying the changes that need to be made to ensure that it is used as standard in usual clinical practice.

The objective of this article is to explore healthcare professionals’ views on CoCPs and the changes that need to be made in an uncontrolled real-life setting. Based on their experiences of ECOPIH and on their points of view, the characteristics that should contribute to the healthcare professionals’ greater readiness to use the CoCP are analysed, as are the changes that need to be made for them to integrate it into their daily clinical practice.

## Methods

### Design

A descriptive-interpretative qualitative study was conducted through interviews of a group of key informants in order to learn about their perceptions of and opinions on the use and usefulness of the ECOPIH platform [36]. A qualitative methodology was appropriate for the purposes of achieving that objective because it enabled deeper knowledge to be gained of the context within which ECOPIH was used and, at the same time, it allowed the professionals’ experiences and perceptions of and reasons for applying that tool to their daily practice to be evaluated [14, 37–39]. Taking into account the discourses of those professionals was essential in order to identify certain aspects that would otherwise be difficult to evaluate using other methodologies, such as social interaction between individuals and how it affects inter-professional collaboration and coordination, as well as the advantages stemming from the platform’s use, and from technological and organisational changes.

### Study setting

The study was conducted on the Barcelonès Nord-Maresme Primary Care Service (PCS) in Catalonia, Spain, which includes 10 PC centres and 3 SC centres (Metropolitana Nord International Health Centre in Santa Coloma de Gramenet, Barcelonès Nord i Maresme Occupational Health Unit in Badalona, and Germans Trias i Pujol University Hospital also in Badalona).

### Participants and selection strategy

PC and SC professionals with communication skills were invited to take part in the study so that they could give comprehensive, in-depth opinions on the ECOPIH tool. They included a majority of users and a minority of non-users of the platform. The sampling method was theoretical, and it included professionals of different ages, professional disciplines (physicians/nurses), positions within the organisation (healthcare or managerial) and role within ECOPIH (participant or consultant). Pragmatic criteria of proximity, accessibility and ease of contact were also taken into account. Discursive representativeness was sought in order to ensure the most comprehensive breadth and depth of information and understanding of the phenomenon, and that is why a combined maximum variation sampling strategy was selected. The principal investigator approached the professionals in their workplace contexts by e-mail to request their participation in the study, looking for the predefined profiles mentioned above. This method offered the advantage of improving contact to request participation and, at the same time, it gave potential candidates greater autonomy to decide whether or not to participate in it. Moreover, we considered that, by having a closer relationship with the principal investigator, those candidates who accepted to participate would be more motivated to give their opinions. It could nevertheless be considered that this very factor might influence the sincerity with which criticism would be expressed. So, at the start of the interviews, special emphasis was placed on the importance of identifying the changes necessary to improve the tool and on the fact that the participants should express themselves with the greatest freedom. The study phenomenon is an innovative topic centred on a tool to facilitate communication between professionals working at different levels of care. Consequently, participation in the study meant that participants had to devote time to the interview in order to recount their experiences of and opinions on ECOPIH, showing critical capacity and interest in improving the tool. Informant selection was considered complete when the categories emerging from the analysis process were saturated. Under those circumstances, incorporating new informants into the study would have meant an unjustified burden for them and a greater analysis workload for the researchers, without providing any significant improvements to the findings [40–43]. A total of 30 professionals were invited to take part by e-mail. Those who accepted signed an informed consent form, which specified that the interviews would be audio recorded. Of those 30, only one person declined to take part because he/she did not want to be recorded. At the end of each session, the participants were offered the chance to receive a copy of the transcription for checking so that the research team could gather feedback from individual informants to assess the validity of findings and ensure that data were interpreted correctly.

### Data-generating techniques

Data was obtained from focus groups, triangular groups and semi-structured individual interviews. These three types of interviews were used because they facilitated the informants’ participation, given their geographical dispersion and other logistical aspects such as time availability for interviews. In addition, the triangular groups allowed topics to be covered in depth with less group pressure, thereby creating more interactive and productive dynamics.

The interviews were held at the centres where the professionals worked to make it easier for them. All the interviews were moderated by the study’s principal investigator. Interview moderation was based on a pre-established topic script that the research team had agreed upon after a review of the literature and a pooling of their experience [see Additional file 1]. In addition, before the interviews started, the moderator stressed the need for the interviewees to express their opinions and experiences of ECOPIH in an honest manner, since the aim was not to obtain polite answers but instead to identify which elements of the tool could be improved. The participants’ characteristics by interview technique type and length are shown in Table 1.

**Table 1:** Characteristics of the participants in the individual and group interviews

	Individual interviews	Triangular group	Focus group	TOTAL
Total number of participants	5	7	17	29
Number of interviews	5	3	2	10
Gender (M:F)	3:2	4:3	4:13	11:18
Age	< 35 years old: 0 35–50 years old: 4 > 50 years old: 1	< 35 years old: 0 35–50 years old: 4 > 50 years old: 3	< 35 years old: 2 35–50 years old: 10 > 50 years old: 5	< 35 years old: 2 35–50 years old: 18 > 50 years old: 9
PC:SC	2:3	2:5	17:0	21:8
Physician: Nurse	5:0	7:0	9:8	21:8
Number of directors	2	5	0	7
Use profile (NU:P:C)*	1:1:3	0:0:7	4:11:2	5:12:12
Length (minutes)	45–60	60–70	90–100	635

^*^Use profile. *NU* Non-user, *P* Participant, *C* Consultant

### Data analysis

Verbatim transcriptions of the recordings were done by the principal investigator, and the informants’ identifying data were anonymised [44]. To aid understanding in this article, quotations from the interviews have been translated into English by a professional academic translator and revised by the research team to verify that the meaning of the original discourse was maintained. The analysis procedures were done manually. A thematic interpretative content analysis [45, 46] was performed and the analysis procedures were done manually by the same investigator. Firstly, the transcriptions were read carefully and repeatedly in order to get an in-depth knowledge and full understanding of them. Such reading enabled pre-analytical intuitions to be developed. In the analysis phase, quotations were identified and coded, and categories were created based on the script of topics explored in the interviews. These were then regrouped and, after analysing each category and establishing relationships, an explanatory framework was finally created. In the case of discourse polarisation (relating to the collaborative virtual environment and to non-access to health records, for example), it was described, analysed and interpreted because it was felt that it offered a relevant point of view. Data collection and analysis was performed in parallel. Thus, as the analysis progressed, the results suggested the acquisition of new data in order to expand and improve the phenomenon’s interpretation, hence the incorporation of new key informants. In particular, an in-depth analysis of the tool’s weaknesses and the proposals for increasing its use was performed. In the analysis phase, the analyst held regular meetings with the research team to discuss and agree on the analysis categories. In addition, the findings were discussed with a researcher outside the project, who was an expert in qualitative research. Quotations from discussions have been included to illustrate the process of interpretation based on data relevance and clarity. The research team remained conscious of their backgrounds and experiences, and how their positionality might influence the analysis and the interpretation of the data. Indeed, the research team was mindful of this throughout every stage of the study and was very clear that the priority was to identify what points could be improved and what changes were needed to ensure that the tool could be incorporated into the context of usual practice within primary care and to contribute to the expected improvements by means of its application, which may have partly controlled for its influence on the results. The research team tried, at all times, to have an ethnographic attitude and to delve into the meanings of the informants’ opinions on and experiences of ECOPIH. In addition, the verbatim transcriptions – and translations thereof – illustrating the data were selected on the basis of criteria of clarity and relevance, and they show the participants’ critical capacity.

## Results

A total of 29 participants were recruited to the study, among whom were 18 women, 21 physicians and 8 nurses. Of the interviewees, 21 were PC professionals, 24 were users of ECOPIH and 7 held managerial positions. A total of 2 focus groups, 3 triangular groups and 5 individual interviews were conducted (Table 1). Additional file 2 shows the profile of each participant.

### Overview

**Fig. 1:**
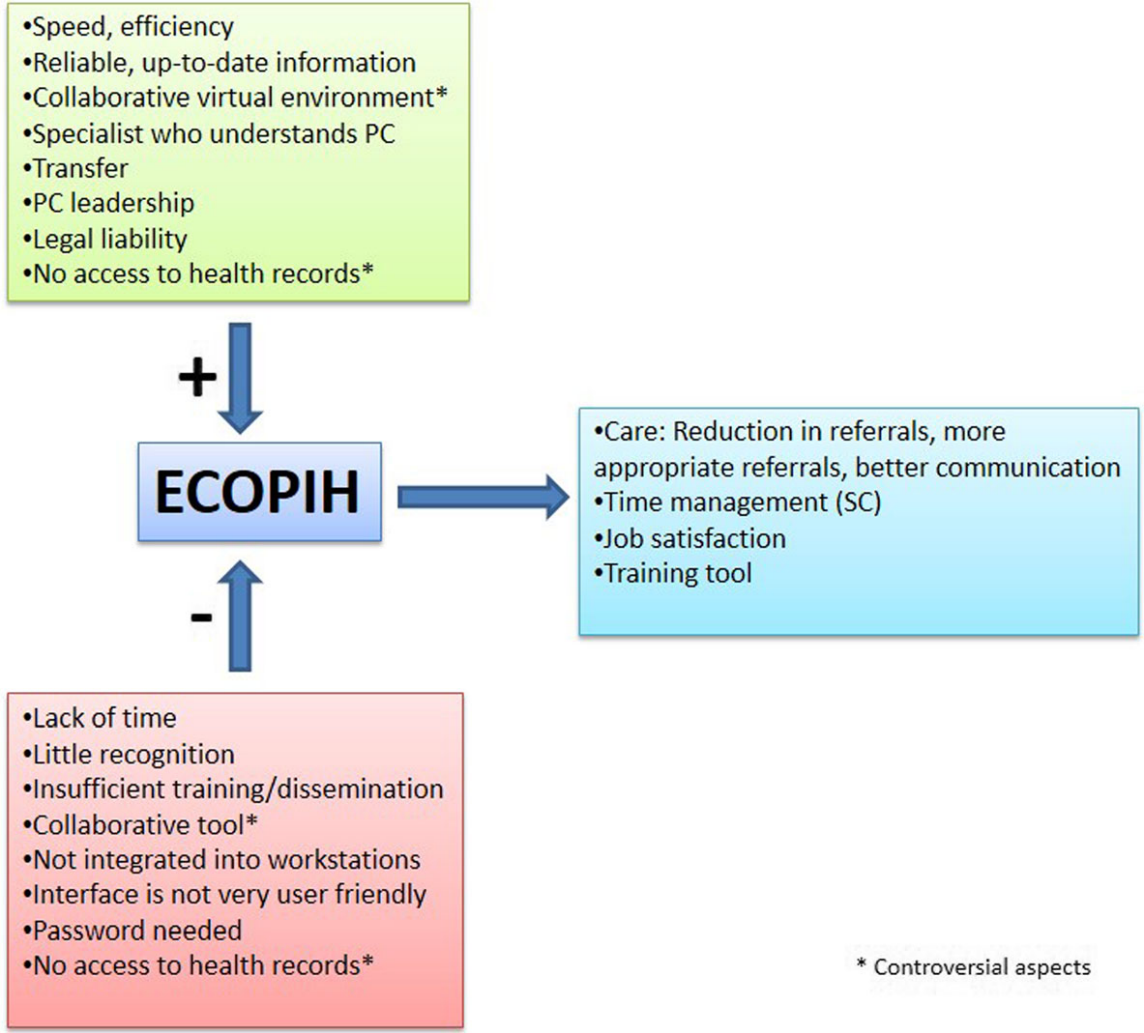
ECOPIH explanatory framework

For a system of communication between PC and SC to become a tool that is habitually used and very useful, the interviewees considered that it should have a series of specific characteristics. Table 2 summarises the key points identified in the interviews and focus groups. The analysis of each of the topics and the relationships among them led to the creation of an explanatory framework of the key points for the platform’s success (Fig. 1).

**Table 2:** What characteristics should a PC-SC communication tool have?

Topic	Key points identified
PC query handling	*“You search for the specialty, you click and then you send it. You don’t need to have any personal contact to get someone to resolve it.” INT. 16 (PC physician, female).* *“I tend to approach the people I work with more (…). I ask the people around me, I think it’s more immediate. I’m quite impatient, so I need immediate answers.” INT. 24 (PC nurse, female, non-user)*
Type of information	*“(The specialists) give you much more comprehensive information than they do when giving an immediate, off-the-cuff answer.” INT. 15 (PC physician, female)* *“It is a reliable source because they are the go-to people.” INT. 19 (PC nurse, female)* *“You may have the clinical practice guide and then you come across patients whose cases fall between the gaps in all of them. The fact that it’s a real patient (in ECOPIH) helps a lot because courses focus mostly on the topic, so then it’s quite hard to adapt it to specific cases that present in the surgery, such as patients with complex conditions. When it comes to providing care, they are real cases that you have to deal with and really need to consult on.” INT. 15 (PC physician, female)*
Knowledge management	*“What we need is a forum where we can discuss things; that would be the ideal clinical session, where you can sit down with your colleagues… that doesn’t happen, or happens very little in the teams. It was the type of tool I needed, that I’d been looking for, and it was good for me.” INT. 4 (PC director, male)* *“When we call the hospital, they answer as fast as they can, as if you were bothering them.” INT. 15 (PC physician, male)* *“Virtual consultation is convenient, provides an answer for that patient and is very powerful. But I still think that they are complementary tools for dealing with knowledge. If I put that query on ECOPIH, I’m asking a more generic question and will find a more generic answer that I can use for other patients too, and thousands of other colleagues of mine will also see it.” INT. 4 (PC director, female)* *“ECOPIH is about building pillars for the future. The other system, virtual consultation, is about improving day-to-day management, the speed of action is much quicker at strategic management level. ECOPIH will give you that in the long term.” INT. 28 (PC director, male)* *“We have quite a few clinical issues to resolve every day over the phone. If more people could see them, perhaps they wouldn’t need to ask about them again. There are many duplicate consultations. That’s the philosophy that needs to prevail.” INT. 2 (SC physician, male)* *“It’s more enriching when everyone can see it, it’s much more enriching for me.” INT. 13 (PC physician, female)* *“The larger the audience, the greater the fear of giving answers; some are undoubtedly a bit more defensive. It has an influence; it curbs the spontaneity that there would otherwise be in certain cases (…). I’m sure it has an influence, and a negative one in some cases.” INT. 8 (SC physician, male)*
Cultural aspects	*“(If ECOPIH had come from the hospital), it would have been used less. Because, if it comes from opposition rather than joint work, it is the hospital that puts its stamp of authority on it, while in primary care they act like automatons within models that may not be the best because there’s been no debate.” INT. 28 (PC director, male)* *“If ECOPIH had come from the hospital, it would have been seen as something quite natural (by the specialists). Instead, it’s something that comes from below, from family doctors. It has created an attitude of anticipation rather than enthusiasm. (…). ECOPIH balances things out, that’s what technologies do, they are very democratic. Here, you treat specialists as equals, but that isn’t understood in the hospital. (…) It’s a change of role. (…) and there’s resistance to change.” INT. 3 (SC director, male)* *“Above all, I think it’s an attitude of wanting to be more proactive, of shaking off your fears and wanting to do things differently.” INT. 2 (SC physician, male)* *“I’d consult more often, but my feeling of embarrassment is quite intense. It’s an insurmountable embarrassment.” INT. 9 (PC physician, female)* *“There aren’t enough nursing topics to consult on because, in nursing, you make your bed and you lie in it. Maybe the direction in nursing is the opposite. In medicine, questions are asked, and in nursing, maybe the experts should be the ones who present news so that people can be informed or debate can be generated.” INT. 23 (PC nurse, female)* *“I find that some of the topics aren’t very specific, there are many medical things.” INT. 19 (PC nurse, female)*
Technological aspects	*“Access needs to be more direct. So that at the time when you have a query about a patient, when its fresh in your mind, you can make it more dynamic.” INT. 15 (PC physician, female)* *“We did have some training, but when you start using it again, you forget what you’ve learned.” INT. 22 (PC nurse, male)*
Organisational changes	*“It’s something connected with work, but when you’re at work you can’t find the time to do it.” INT. 19 (PC nurse, female)* *“It depends a lot on how you understand your profession. If you’re curious and need to increase your knowledge, you’ll use ECOPIH or you’ll study at home, you have to read, you need time.” INT. 4 (PC director, female)* *“If you link it to senior management, you might undermine the tool to some extent because it is perceived as a form of managerial control. When MBO ends, the tool ends because there hasn’t been any personal motivation. It’s risky, it might by counterproductive for the tool.” INT. 28 (PC director, male)* *“Recognition of the tool itself by provider companies is what’s missing; they need to integrate it into the initial visits. The two managers, of primary care and hospital care, need to sit down and decide what it means, how to recognise this work.” INT. 3 (SC director, male)*
Legal liability	*“I understand that it’s a secure tool. It doesn’t worry me, I do things that are much more insecure than this, for example, replying by e-mail, a telephone call… The thing here, though, is that it’s in writing, and it stays that way forever. The legal ramifications of this don’t worry me, but the very lack of definition of project makes me wonder: ‘Here, if I make a mistake and I receive a complaint about something I’ve said here, would the Catalan Health Institute consider it theirs?’.” INT. 3 (SC director, male)*

### PC query handling

Many aspects of ECOPIH were valued positively. A considerable number of the interviewees highlighted the effectiveness and speed of responses, as well as the ease of accessing the platform and contacting a non-GP specialist. This speed (responses within 24–48 h) might not be quick enough for some members of the nursing group, who are used to the immediacy of consulting with colleagues nearest to them as a way of resolving queries (Table 2).

### Type of information that people want to find

The information obtained from the ECOPIH query was valued positively by the interviewees because they considered it comprehensive, reliable and up to date.

On the other hand, some professionals mentioned the common difficulty of finding information that is directly applicable to real PC patients, because such patients are often under-represented in clinical practice guides or training courses. Clinical cases specific to PC patients were found in ECOPIH, thus facilitating the transfer of advice given by the non-GP specialists to clinical practice.

Several participants (INT. 9, 16, 26) mentioned that this aspect improved when the consultant non-GP specialist was a professional who had an understanding of PC and was close to it, since there were greater similarities in patient focus. In addition, a more personal relationship could be established, thus increasing trust in the answer (Table 2).

### Knowledge management

In the interviews, the professionals identified the need to establish an approach that would bring different areas of care closer together in order to achieve a collaborative working culture that would benefit patients.

Different communication mechanisms between care levels have traditionally been set up. Despite that, the participants in our study stated that contact via these channels of a more classic nature had major limitations. Thus, the telephone channel presented the difficulty of locating the non-GP specialist and, when located, he/she did not often show the necessary predisposition, or perhaps the moment was not right for such interaction, which he/she experienced as an interruption. Classic face-to-face consultation also had physical and temporal constraints (limited timetables, a lack of recording for subsequent consultations, limited information conveyed to both professionals), which forced anyone who wished to raise a query with a non-GP specialist to do so through personal contacts in informal conversations (“cronyism”) or by interrupting clinics.

Regarding the coexistence of ECOPIH alongside other SC communication systems, whether traditional (e.g., face-to-face consultations) are more innovative (e.g., virtual specialist consultations through access to patients’ health records), several interviewees highlighted that, without underestimating the obvious advantages of such systems, the consultations of clinical cases raised via ECOPIH were more interesting and comprehensive, and that they added educational value for future cases. The tools were therefore complementary.

Several participants explained that such communication should ideally take place virtually. They also said that it would be positive if it became a common forum for sharing clinical cases. For most of the interviewees, the fact that content was visible to all users, who in turn could get involved in the discussion about the case, was an advantage. They also felt that it should be used more widely, so long as patient confidentiality was assured. This form of consultation was enriching because it allowed people to learn about other ways of working and helped to reassure professionals who could see that other colleagues had the same queries as they did. The transmitted knowledge was spread widely and learning was fostered. This was also highlighted by the non-GP specialists, who considered that many of the consultations they received via e-mail were similar. Thus, a public discussion forum would prevent duplicate consultations.

Despite that, a few professionals considered that creating debates on consultations was not positive and that they should stay within a question-answer system. They also felt that tense situations might be created if there was disagreement with the non-GP specialist. Some PC users believed that the collaborative aspect of the tool might represent a barrier because some professionals are reluctant to express themselves in public, because it might lead to doubts about who has access to the content (management, senior management, etc.) and because it might make the consultants’ answers more defensive (Table 2).

### Cultural aspects

The interviewees mentioned the effect of the tool’s origin on predisposition towards its use. Thus, the PC professionals said that they would have been more receptive if the tool had been created within PC itself, which would have led to greater use. SC had remained expectant for the same reason.

On the other hand, some professionals interviewed considered that one of the main determining aspects of ECOPIH use was people’s attitudes towards such tools. Thus, qualities like being receptive to new ideas, proactivity, enthusiasm and predisposition towards sharing doubts in a group situation were essential, and the lack thereof partly explained a low level of participation. They also highlighted the importance of shaking off one’s fears of embarrassment when expressing one’s doubts in public, though they also mentioned that this concern could be resolved in part if the consultations were anonymous.

On this point, it should be noted that, according to some of the interviewees, the nursing group was less accustomed to sharing their doubts and therefore adopted a passive attitude more often. Together with the fact that other users felt that few specific nursing-related topics were mentioned, this aspect meant that the nursing group’s use of the tool was lower (Table 2).

### Technological aspects

Regarding the technological aspects, the main problem that users referred to was the high number of platforms and workstations used in daily practice, each with its own particular characteristics: electronic health records, shared health records, e-mail, other consultation systems, etc. The use of ECOPIH via the e-Catalunya platform did not present any major difficulties other than the personal skills of each user of the technology. That said, it seemed to be a platform that was not sufficiently easy to use because it required too many clicks and intermediate steps to get to the consultations. Older users found the platform more difficult to handle, and personal attitude was again the key factor in overcoming that limitation.

Regarding the e-Catalunya platform, several professionals suggested the following improvements: making access easier by integrating it into habitual workstations or a mobile app; grouping information by topic given that a lot of content was building up over time; having an e-mail notification system containing the text of new contributions; and having the option to use filters or lists of the most frequent consultations.

Regarding the possibility of accessing the patients’ health records, the participants felt that, while it would make the tool more formal, it could jeopardise its immediacy and ease of use, and would introduce conflicts with respect to confidentiality and disseminating knowledge among other users.

Although the e-Catalunya platform’s operation was straightforward for most users, some professionals needed more ongoing support or training to maintain their skills in order to use the tool (Table 2).

### Organisational changes

One of the main determining factors of ECOPIH non-use was the lack of available time during the working day, despite the fact that the tool was wholly connected with work. Most of the interviewees rejected the idea of spending their own time on this task, although some considered that learning and trying to resolve unclear cases in their own time was part and parcel of the profession.

On the other hand, the interviewees highlighted the little or zero recognition of the activities that they undertook in ECOPIH by the senior management of the various services or provider companies. As the platform evolved, someone suggested including its use as an evaluation indicator in the Management by Objectives (MBO) appraisal [47] in order to foster and recognise its use. It was a controversial topic because, while some people did consider it a good strategy to increase its use, most of the users were against that option because it might cause rejection due to it being interpreted as something connected with senior management.

In short, several users mentioned that the organisation should decide whether or not it wanted roll out a tool like ECOPIH, reflecting that commitment in a contract or agreement to enable the associated tasks to be included in the service portfolio of each specialty. Thus, the work done could be recognised by counting it as if it were a referral for consultation (Table 2).

### Legal liability

Finally, all of the PC professionals interviewed were very clear about the fact that legal liability would fall to the PC professional dealing with the patient because that was how it was stated in the ECOPIH usage rules. The non-GP specialists taking part in this study were not concerned about this issue because they considered that the opinion expressed was advice, corresponding to the non-GP specialists’ theoretical action as it would have been taken in a consultation. They highlighted that they habitually undertook other consultation activities as if they were referrals, such as telephone calls and e-mails, which were rather more insecure in legal terms. Nevertheless, a few interviewees did express certain doubts about the legality of these types of actions because the answers were recorded in writing, as was the reply from the institution in the event of a complaint (Table 2).

### Benefits derived from using ECOPIH

According to the interviews conducted, the use of ECOPIH offered a series of professional and organisational benefits (Table 3). To begin with, most of the interviewees considered that ECOPIH reduced the number of referrals for two reasons. Firstly, it enabled the doubt that would usually have led to a referral to be resolved, and secondly, it allowed the professionals to read the cases raised beforehand, thus leading to a better handling of patients in PC and, therefore, a reduction in the number of referrals and visits to SC. Many of the participants explained that, besides referring fewer patients, any referrals that were made would be more appropriate. The professionals therefore considered ECOPIH an efficient tool.

**Table 3:** Benefits derived from using ECOPIH

*“Of course, it reduced referrals. You refer when you have doubts about how to handle a case and you want to seek the opinion of an expert in another topic. If you manage to get this information by other means and you end up handling the case while counting on the non-GP specialist’s support at all times, ultimately you don’t refer it.” INT. 16 (PC physician, female).* *“If you come across a case that has already been discussed, you don’t refer it because you have the answer.” INT. 13 (PC physician, female).* *“It could prevent referrals that are sometimes unnecessary. (…) It should save on visits and that means saving money and duplicate tests, so it could be an efficient tool.” INT. 26 (SC physician, female).* *“It isn’t a huge reduction in referrals, it’s referring properly… and learning.” INT. 9 (PC physician, female).* *“It’s efficient in the sense that the patient doesn’t have to go from one place to another. It prevents silly consultations from getting onto the waiting list (…) and has the potential to improve the care offered to the patient. It’s a good tool, it’s useful and relevant, it’s safe, it has the ability to resolve issues and is probably efficient, although I can’t assure you of that. It’s a new way of operating that, if it saves work, will ultimately make us more efficient.” INT. 3 (SC director, male).* *“I think ECOPIH improves the quality of care the patient gets. It helps me and my patient.” INT. 4 (PC director, female).* *“It does reduce referrals a little, and if it doesn’t, it means they are made properly. Sometimes it isn’t as much as saying ‘refer it to me’ as saying ‘refer it to me, but do this’ or ‘refer it to me with this priority’. It provides clarification when it comes to having to refer or not.” INT. 29 (SC physician, female).*	

Even those interviewees who occupied managerial positions felt that it was an efficient tool that could reduce waiting lists, especially when a referral could not be justified for any particular reason.

There was more consensus among the users about the fact that the tool improved the quality of the referrals, for several reasons. It prevented inappropriate referrals and increased higher quality referrals. In addition, they were clinical cases in which more thought and work had been invested, so much so that on a few occasions, the simple fact of preparing a case for consultation in ECOPIH had enabled it to be resolved. Finally, for those cases that were discussed and ultimately referred, a higher number of supplementary tests had already been done. In other words, even though the response to the cases raised was to refer them, such referral was done in a more appropriate and timely way.

### Overall appraisal of the tool

Several PC physicians stated that that they were happy when they had time to raise cases for consultation on the platform, were relieved when they obtained solutions to their queries, were confident about how to handle patients and, finally, were satisfied with the profession itself because they had managed to achieve optimum handling of particular cases in an independent manner.

Most of the non-GP specialists also referred to this increase in satisfaction, and they also considered that ECOPIH was a useful tool for improving time management because it led to less interference in daily activities at work than other consultation systems, e.g., the phone (Table 4).

**Table 4:** Overall appraisal of the tool

*“You have a query and, before you know it, you find it (the solution) right there and you’re really relieved, you resolve it straight away, you learn, you sort it out.” INT. 15 (PC physician, female).* *“It’s a trustworthy tool for family doctors.” INT. 14 (PC physician, male).* *“Satisfaction with the profession itself because, apart from experience that gives you time, what we need to do is increase our own knowledge.” INT. 4 (PC director, female).* *“ECOPIH helps to manage time. It removes the urgency of demands. It’s the best way to manage time, knowing what you’ve got in front of you and being able to decide on the right order of execution.” INT. 8 (SC physician, male).* *“For us, being in ECOPIH is a strength. It’s a way of becoming visible. It has helped to break down barriers, to bring professionals closer together, and that will also have reinforced its use. It’s a very positive tool for improving communication among the entire community of physicians.” INT. 8 (SC physician, male).* *“Creating feedback between primary and non-GP specialist care is unbeatable, I think the idea is really great.” INT. 29 (SC physician, female).* *“It has the educational aspect that a virtual visit doesn’t have, and it has the care aspect too, depending on how you apply that to a particular case while working. For a physician, training and practice are one and the same thing. If you’re well-trained, your practice is better, if you’re practice is better, you work better with your patients, that means everything, referrals, etc.” INT. 28 (PC director, male).* *“People who regularly go into ECOPIH… after one or two years those people know a lot more, if they’re active, than people who’ve done courses on goodness knows what. (…) It is a much more pragmatic, clear and practical kind of training because you can apply it straight away and can improve care.” INT. 28 (PC director, male).* *“It’s expert learning, it’s case-based learning because your learning from the case (…).” INT. 11 (PC physician, female).* *“I can see a lot of advantages in it for learning, I’m surprised by how much I manage to learn. It would be an à la carte continuing medical education because you can choose the topic.” INT. 12 (PC physician, female).*	

From the SC point of view, participation in ECOPIH was valued positively in the majority of cases because it was considered a tool that enabled communication between care levels to be improved.

Most of the interviewees considered that ECOPIH fulfilled both the educational and the care functions. This was due in part to the fact that the knowledge acquired (education) became applicable to clinical practice (care), thus implying that they were two interrelated concepts.

Some participants clarified that raising cases for consultation would mostly have a care-related function because it generally enabled real clinical cases to be resolved so long as answers were given quickly. They nevertheless felt that it would also have an educational function for other members reading the consultations made. In contrast, they considered that sharing documents would basically have an educational function. In addition, the non-GP specialists saw other advantages, such as identifying training needs for PC and even the fact that the tool could become a means of accessing self-learning in SC.

Several interviewees highlighted that ECOPIH came across as a powerful training tool, mainly because learning was based on real cases, was much more pragmatic and was directly applicable to clinical practice or to similar future cases. A few participants added that having many specialities available for consultation made it a kind of à la carte continuing medical education. It should be noted that it was pointed out on several occasions that acting solely as an observer or reader of content could increase learning.

## Discussion

The need for healthcare professionals to be able to access trustworthy sources of information is well known, as is the fact that scientific literature may not be able to give direct answers to clinical questions arising in daily practice [7, 11, 48]. Having a tool that provides quick, practical and reliable information is essential for PC professionals given the multitude of queries arising in daily clinical practice [2, 6, 8, 49–51]. The opinions of ECOPIH users showed that the tool managed to meet that need. The fact that consultant non-GP specialists knew about the context within which AP physicians worked made a definitive contribution to that, and even more so if they were the go-to professionals for the PC area from which patients were referred.

According to the participating professionals, the gap between PC and SC could be bridged by implementing virtual communication tools [52–54]. ECOPIH includes the Web 2.0 concept in communication among professionals, and it does so via a CoCP, thereby triggering a change in how knowledge is managed. CoPs provide a useful model for knowledge management as well as a mechanism that facilitates and promotes a new way of working and learning based on collaborative working and the use of collective intelligence [55]. They can be especially useful in PC, where flexibility and constant coordination are key aspects of caring for patients with significant multimorbidity [56, 57].

Not surprisingly, the ECOPIH tool has a very powerful educational component that combines four aspects that, in our opinion, are essential: peer learning with the presence of an expert [32–34, 58]; learning based on real clinical cases that is directly applicable to clinical practice [20, 21]; the dissemination of knowledge to the entire community [59] (even without any active participation, i.e., lurkers); and social interaction, which is one of the main channels through which healthcare professionals create their own tacit knowledge [19, 60–64]. As in any CoP, recently qualified physicians learn by interacting with experts, who in turn may acquire new skills. In addition, collective knowledge is created and becomes available to the community over time [58, 65, 66]. They also learn together by focusing on problems that are directly connected with their work, and this is something that increases the participants’ motivation, since their learning is linked to problematic situations that they can recognise or perceive as real and applicable to their work [20–22]. This is particularly relevant because the tool provides a framework for the professional development of individuals in the workplace through different forms of participation [67–69]. Hence, the ECOPIH platform offers advantages from the points of view of care and education because its use is not limited to resolving specific cases. The non-GP specialists’ advice and the literature attached to it also enable other colleagues to resolve similar cases. The accumulation of experience increases not only the group’s explicit knowledge, but also its tacit or practical knowledge, which emerges from reflective practice and from gathering and sharing cases among professionals [56].

On the other hand, it is also worth noting ECOPIH’s considerable usefulness as time management tool for SC professionals, since it allows them to decide on how much time to spend on communicating with PC by avoiding interruptions and duplicate consultations. However, the participants often identified the lack of time as the main determining factor of ECOPIH use for resolving queries. The use of virtual communication tools like ECOPIH requires organisational changes to allow PC and SC professionals to have that time available regularly. Although participation in a CoP occurs partly because it has a certain value for the users, irrespective of whether or not it is an institutional directive [59, 70, 71], an institution must commit to the tool by incorporating it into its service portfolio [72] and by giving recognition to participants in general and to consultants in particular [20]. However, the latter issue must be addressed with care because certain incentives to use it, such as MBOs, may represent a barrier to its use. In fact, despite the implementation of networked clinical structures aimed at improving patient care and facilitating knowledge-sharing among healthcare professionals beyond the boundaries of organisations, certain bureaucratic, hierarchical and intra-professional barriers may still exist [73]. On the other hand, the time barrier could be overcome through training [74, 75], the promotion of the tool’s potential usefulness, high-value content and improved technological aspects [24, 58]. More research in this field is required [76].

This point ties in with the technological issues. Ideally, the platform should be integrated into habitual workstations without the need to enter a new password [74, 77–79] and the interface should be user friendly [80]. The platform should also have a series of technical features that make it easy to use (information search and filter functions, a mobile app, etc.) [11, 48, 49, 81]. While there is some controversy surrounding the issue, it is generally considered unnecessary to have access to the patients’ health records, mainly to ensure that ECOPIH’s ease of use is not compromised. Nevertheless, if it were technically possible, it might be interesting to have an optional link to access patients’ health records in specific instances, so long as such access is password protected and contained within a secure environment.

Concerns about legal liability stemming from advice given by a non-GP specialist via an online app have been identified in several studies, especially among SC members [15, 80, 82–86]. While virtual consultations are often considered informal, a number of peculiarities make the legalities surrounding them somewhat complex, so this is something that must be reviewed before the implementation of an online CoCP. In the case of ECOPIH, this was not a contentious issue because it was made very clear, in writing and right from the start, that the responsibility for the patients’ care fell to the PC professionals; this was made explicit during the training and was visibly very prominent on the tool itself.

According to the scientific literature, a key factor for the success of a telemedicine project is that clinical professionals should be responsible for its leadership [38, 80, 87]. In our case, the experience was led by a PC professional who had a perfect understanding of the reality of professionals in that sphere and of their needs. This enabled him to adapt the tool technically and organisationally. All of this entails a series of intangible benefits, which were identified on numerous occasions in the interviews conducted, as well as in previous studies [55, 88, 89]. By improving communication between care levels, greater peace of mind and confidence when handling patients is achieved [90, 91]. This leads to improved job satisfaction for PC and SC professionals [67, 92]. According to the users of this CoCP, ECOPIH helped them reduce the number of referrals and make them more appropriate [84, 93]. However, in order to evaluate every dimension of the tool, further research should be done from a qualitative perspective on the impact of CoCPs in financial terms (a reduction in referrals and visits, and cost analysis) and clinical terms.

The rigorous procedures used (a detailed description of the context and the participants, the reflexivity of the research team and the theoretical sample to achieve discourse saturation) ensure the validity of the findings in our setting. However, caution is needed before transferring these results to other settings. In this respect, the sample selected in our study also took into account pragmatic criteria such as access to the interviewees, hence the discourses of professionals working in a rural setting were not taken into consideration. Nor was the possible effect of the population’s socioeconomic position, which might have an influence on professional practice, on workload and on the information requested by citizens, thereby modifying ECOPIH use.

## Conclusions

In the healthcare sphere, inter- and intra-organisational networks are crucial to the creation and dissemination of clinical knowledge because such knowledge is experiential, implicit or tacit [93]. The ECOPIH platform has proved to be a useful, satisfactory tool for improving the healthcare provided by PC professionals. It stands out as a collaborative tool that provides reliable, up-to-date information that is highly transferrable to clinical practice. The users valued its effectiveness, efficiency and educational capacity, and they considered that it improved job satisfaction. In order to make the most of its potential in terms of care and education*,* organisational changes are required to free up sufficient time for participants to access the tool habitually (whenever needed), as well as cultural changes for knowledge-sharing and networking, and technological changes linked to the platform and to its integration into the healthcare professionals’ habitual workstations.

## Additional files

Additional file 1: Table with the interview topic script developed by the research team. (DOC 45 kb)

Additional file 2: Table with the Interviewees’ profiles of the 29 participants recruited to the study. (DOC 38 kb)

## Abbreviations

CoCP: Community of clinical practice
CoP: Community of practice
ECOPIH: Eina de Comunicació entre Primària i Hospitalària (Online Communication Tool between Primary and Hospital Care)
ICT: Information and communication technologies
PC: Primary care
PCS: Primary Care Service
SC: Non-GP specialist care
UOC: Open University of Catalonia
