# Correction to: Keys to success of a community of clinical practice in primary care: a qualitative evaluation of the ECOPIH project

**DOI:** 10.1186/s12875-020-01132-x

**Published:** 2020-04-17

**Authors:** David Lacasta Tintorer, Josep Maria Manresa Domínguez, Enriqueta Pujol-Rivera, Souhel Flayeh Beneyto, Xavier Mundet Tuduri, Francesc Saigí-Rubió

**Affiliations:** 1 Centre d’Atenció Primària Gran Sol, Gerència d’Àmbit d’Atenció Primària Metropolitana Nord, Institut Català de la Salut, Avinguda del Doctor Bassols, 112 - 130, 08914 Badalona, Spain; 2 Unitat de Suport a la Recerca Metropolitana Nord, IDIAP Jordi Gol. CAP El Maresme, Camí del Mig, 36 planta 4a, 08303 Mataró, Spain; 3 Departament de Medicina, Universitat Autònoma de Barcelona, Plaça Cívica, s/n, 08193 Bellaterra, Cerdanyola del Vallès, Spain; 4 Institut Universitari d’Investigació en Atenció Primària (IDIAP Jordi Gol), Gran Via Corts Catalanes, 587, àtic, 08007 Barcelona, Spain; 5 Unitat de Suport a la Recerca Barcelona Ciutat, IDIAP Jordi Gol, Carrer Sardenya 375, 08025 Barcelona, Spain; 6 Faculty of Health Sciences, Universitat Oberta de Catalunya, Barcelona. Av. Tibidabo, 39-43, 08035 Barcelona, Spain

**Correction to: BMC Fam Pract (2018) 19:56**

**https://doi.org/10.1186/s12875-018-0739-0**

Following publication of the original article [1], the authors opted to update affiliation 3 in order to comply with the current regulations for the submission of Doctoral Thesis by compendium of articles, the Universitat Autónoma de Barcelona Doctoral School asks us to update the affiliation number 3, adding “Departament de Medicina” at the beginning, as follows:

3 Departament de Medicina, Universitat Autònoma de Barcelona, Plaça Cívica, s/n, 08193 Bellaterra, Cerdanyola del Vallès, Spain.
