# Supplementary material for: Keys to success of a community of clinical practice in primary care: a qualitative evaluation of the ECOPIH project
Source: BMC Fam Pract. 2018 May 9;19:56. doi: 10.1186/s12875-018-0739-0 (PMC5944103; doi:10.1186/s12875-018-0739-0)
Supplement: Supplementary file 1 — Table with the interview topic script developed by the research team. (DOC 45 kb) [file 12875_2018_739_MOESM1_ESM.doc]

**Additional file 1. Interview script**

| TOPICS | QUESTIONS |
| --- | --- |
| **Use of 2.0 tools** | - Do you habitually use social media websites like Facebook, Twitter, blogs, etc.? - If not, why not? - What communication advantages do you think these social media websites have? - Do you use any of these social media websites at work? With patients? For training? To provide care? Why? - What work-related advantages and disadvantages do you think the use of these social media websites has? |
| **ECOPIH dissemination** | - How did you find out about ECOPIH? - Do the professionals you work with know about this tool? |
| **ECOPIH use** | - Do you use ECOPIH? - Why do you use ECOPIH? - Why don’t you use it? - At what time of the day do you usually use it? |
| **ECOPIH usefulness** | - Do you think ECOPIH is more useful for care or for education? |
| **Need for the tool** | - Do think it’s necessary for there to be a virtual tool like ECOPIH to consult specialists? |
| **Positive aspects of ECOPIH** | - What are the positive aspects of the tool? - What do you think the advantages of using ECOPIH are? |
| **Negative aspects of ECOPIH** | - What are the negative aspects of the tool? - What difficulties associated with the ECOPIH’s use can be put down either to the tool itself or to the organisation? - Do you think the potential liability of statements made or opinions expressed is a concern for consultants, despite the fact that ECOPIH’s usage rules clarify this issue? |
| **Training on the tool** | - Do you think the training you received on the tool was sufficient? Why? What did you find missing? |
| **Impact on referrals** | - How do you think ECOPIH use might affect referrals? Could it reduce the amount? Or increase it? Could it improve their quality or make them more appropriate? Why? Why do you think this effect is not seen in the quantitative analysis? - Are the cases discussed representative of the cases seen in primary care (PC) surgeries? - Was the level or complexity of consultations what you expected? |
| **Patients’ perspective** | - Do your patients know that you raise cases for consultation in ECOPIH? Why? Do you think it’s good that they know? - What do you think their opinions are or might be? - Do you think ECOPIH use can improve healthcare from the patients’ perspective? How? |
| **Impact on professionals’ satisfaction** | - Do you think ECOPIH use improves healthcare professionals’ job satisfaction? |
| **Impact on communication between care levels** | - Do you think ECOPIH use improves communication between PC and SC professionals? Why? - And among PC professionals themselves? Why? |
| **Web 2.0 concept** | - What advantages do you think there are in being able to see other colleagues’ consultations? What disadvantages are there? - Do you think it’s better to use tools that keep conversations with specialists in a private sphere? - What are your views on the fact that different specialties might resolve the same case? - What value do you attach to the fact that other PC colleagues can help to resolve clinical cases? |
| **Aspects for improvement** | - How do you think the tool could be improved to ensure that it is used more? - What do you think the future of a tool like ECOPIH is? |
| **Other comments** | - Would you like to add any other comments? |
