# Supplementary material for: Keys to success of a community of clinical practice in primary care: a qualitative evaluation of the ECOPIH project
Source: BMC Fam Pract. 2018 May 9;19:56. doi: 10.1186/s12875-018-0739-0 (PMC5944103; doi:10.1186/s12875-018-0739-0)
Supplement: Supplementary file 2 — Table with the Interviewees’ profiles of the 29 participants recruited to the study. (DOC 38 kb) [file 12875_2018_739_MOESM2_ESM.doc]

| **Additional file 2. Interviewees’ profiles** | | | |
| --- | --- | --- | --- |
| Interviewee number | Profile | Interviewee number | Profile |
| INT. 1 | Male, physician, 35-50 years old, specialist, user | INT. 16 | Female, physician, 35-50 years old, PC, user |
| INT. 2 | Male, physician, 35-50 years old, specialist, user | INT. 17 | Female, physician, > 50 years old, PC, non-user |
| INT. 3 | Male, physician, 35-50 years old, specialist, director, user | INT. 18 | Female, nurse, 35-50 years old, PC, user |
| INT. 4 | Female, physician, > 50 years old, PC, director, user | INT. 19 | Female, nurse, 35-50 years old, PC, user |
| INT. 5 | Male, physician, > 50 years old, specialist, director, user | INT. 20 | Male, nurse, > 50 years old, PC, consultant |
| INT. 6 | Male, physician, > 50 years old, specialist, director, user | INT. 21 | Female, nurse, 35-50 years old, PC, user |
| INT. 7 | Female, physician, > 50 years old, specialist, director, user | INT. 22 | Male, nurse, 35-50 years old, PC, consultant |
| INT. 8 | Male, physician, 35-50 years old, specialist, user | INT. 23 | Female, nurse, 35-50 years old, PC, user |
| INT. 9 | Female, physician, > 50 years old, PC, user | INT. 24 | Female, nurse, > 50 years old, PC, non-user |
| INT. 10 | Male, physician, > 50 years old, PC, non-user | INT. 25 | Female, physician, 35-50 years old, PC, user |
| INT. 11 | Female, physician, > 50 years old, PC, user | INT. 26 | Female, physician, 35-50 years old, specialist, user |
| INT. 12 | Female, physician, 35-50 years old, PC, user | INT. 27 | Female, physician, 35-50 years old, specialist, user |
| INT. 13 | Female, physician, 35-50 years old, PC, user | INT. 28 | Male, physician, 35-50 years old, PC, director, user |
| INT. 14 | Male, physician, 35-50 years old, PC, user | INT. 29 | Female, physician, 35-50 years old, specialist, user |
| INT. 15 | Female, physician, < 35 years old, PC, user |  |  |
